# Tunable Antireflection Properties with Self-Assembled Nanopillar and Nanohole Structure

**DOI:** 10.3390/nano12244466

**Published:** 2022-12-15

**Authors:** Tangyou Sun, Furong Shui, Taohua Ning, Wenjing Guo, Zhiping Zhou, Zanhui Chen, Cheng Qian, Qian Li

**Affiliations:** 1 Guangxi Key Laboratory of Precision Navigation Technology and Application, Guilin University of Electronic Technology, Guilin 541004, China; 2 State Key Laboratory of Advanced Optical Communication Systems and Networks, School of Electronics Engineering and Computer Science, Peking University, Beijing 100871, China; 3 PerkinElmer Management (Shanghai) Co., Ltd., Shanghai 201203, China; 4 Synergetic Innovation Center for Quantum Effects and Application, Key Laboratory of Low-Dimensional Quantum Structures and Quantum Control of Ministry of Education, College of Physics and Information Science, Hunan Normal University, Changsha 410081, China

**Keywords:** antireflection, self-assembly, polystyrene spheres, Finite-Difference Time-Domain, photoelectronic devices

## Abstract

Nanostructure engineering has proven to be one of the most effective strategies to improve the efficiency of photoelectric devices. Herein, we numerically investigate and experimentally demonstrate a self-assembled silicon-based nanopillars and nanoholes structures, to improve the light absorption of photoelectric devices by an antireflection enhancement. The nanopillars and nanoholes structures are fabricated by the air–liquid interface self-assembly method based on polystyrene (PS) nanospheres. Additionally, the tunable antireflective properties with the different operation wavelength and nanostructures parameters have been discussed based on the Finite-Difference Time-Domain (FDTD) method. The experimental result shows that the self-assembled silicon-based nanopillars and nanoholes structures can achieve the lowest reflectivity of 1.42% (nanopillars) and 5.83% (nanoholes) in the wavelength range of 250–800 nm, which reduced 95.97% and 84.83%, respectively, compared with the plane silicon. The operation mechanism of the tunable antireflective property of self-assembled nanopillars and nanoholes structures is also analyzed in the simulation. Our study suggests that the self-assembled nanopillars and nanoholes structures are potentially attractive as improving efficiency of photoelectric devices.

## 1. Introduction

Photoelectric conversion efficiency is crucial to the evaluation of quality in photoelectronic devices, and improving efficiency has been spurring researchers to seek alternatives to this problem. One of the effective methods is enhancing the light–matter interaction with the substrate material. As well known, silicon as a substrate material is widely used in optoelectronic devices. Nevertheless, the high reflectivity of silicon surface is still a prominent problem for light absorption capacity [1,2,3], which results in the low efficiency of photoelectric devices [4,5,6,7,8]. Up to now, a variety of photoelectric devices has applied the antireflective technology to improve efficiency, such as solar cells [9,10,11], LEDs [12,13,14], photoelectric detections, and sensors [15,16]. Nanostructure engineering has proven to be a promising strategy for the antireflective methods [17,18,19], such as nanopyramids [20,21], nanorods [22,23], and nanopillars [24]. Nanostructure not only can reduce the high refractive index difference between air and silicon, but also promote more scattered light into the material and restrain more reflected light, which greatly benefits the improving efficiency of photoelectric devices [25,26]. Therefore, an abundance of nanostructure assembly methods have emerged [27,28,29,30,31]. In addition, some conventional lithographic techniques have been demonstrated for the fabrication of assembled nanostructures, such as electron beam lithography (EBL) [29,32], ion beam lithography (IBL) [30], X-ray lithography (XRL) [33,34], and photolithography [35]. However, these techniques have their unavoidable limits, such as high cost per nanostructure, time-consuming, low sample throughput, complicated multi-step, which restrict their applications in some common laboratory situations.

More recently, self-assembly method based on the polystyrene (PS) nanospheres has been putting into the low cost and large-area fabrication in common laboratory situations, due to the advantages of a high-quality, low cost and large-area template monolayer mask [36,37]. By combining with the reactive ion etching (RIE) and evaporation technologies, a two-dimensional periodic diffraction grating nanostructures with controllable size can be produced massively. In the experiment, the parameters of nanostructures cannot be adjusted easily because of the limitations on the number and the cost of experiments. To optimize the parameters of nanostructures, a simulation was also applied after the experiment.

In this paper, we experimentally demonstrate and numerically investigate the self-assembled silicon-based nanopillars and nanoholes nanostructures, for which increase anti-reflection efficiency by applying the nanostructure engineering on the Si substrate. Unlike the typical lithographic techniques, self-assembled nanopillars and nanoholes structures were fabricated in low cost and large-area by the air–liquid interface self-assembly method based on PS nanospheres. The self-assembled silicon-based nanopillars and nanoholes structures can achieve the lowest reflectivity of 1.42% (nanopillars) and 5.83% (nanoholes) in wavelength range of 250–800 nm, which reduced 95.97% and 84.83%, respectively, compared with the plane silicon. Additionally, the simulated results based on the finite-difference time-domain (FDTD) method matched well with the experiment data. In the meantime, the tunable antireflective properties with different operation wavelength and nanostructures parameters have been found. Furthermore, the tunable antireflective property can be attribute to the diffraction phenomenon of self-assembled nanopillars and nanoholes structures, which is proven closely relevant to the parameters of nanostructures and wavelength in the simulation. Our research suggests that the self-assembled nanopillars and nanoholes structures can potentially increase efficiency of photoelectric devices.

## 2. Experimental

### 2.1. Fabrication of Nanopillars and Nanoholes Structures on Si Substrate by the Air–Liquid Interface Self-Assembly Method

**Figure 1:**
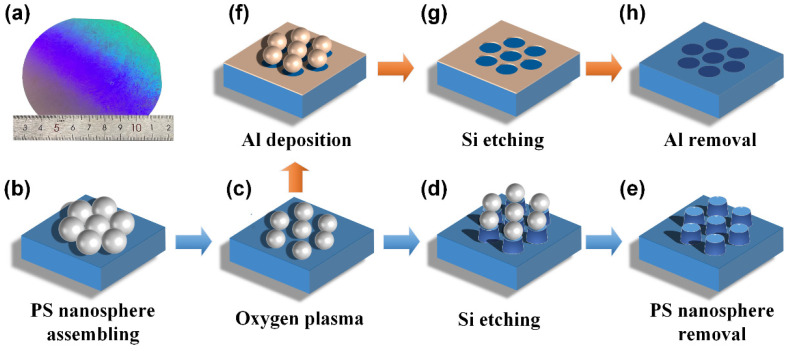
The schematic diagram of nanopillars and nanoholes experimental flow based on the air–liquid interface self-assembly method: (**a**) The sample of PS nanospheres template mask on Si substrate, (**b**) PS nanospheres assembling, (**c**) oxygen plasma, (**d**) Si etching, (**e**) PS nanospheres removal, (**f**) Al deposition, (**g**) Si etching, (**h**) Al removal.

Figure 1 shows the schematic diagram of nanopillars and nanoholes experimental flow based on the air–liquid interface self-assembly method. The sample of PS nanospheres template mask on Si substrate as shown in Figure 1a. The PS nanospherical latex solution with a diameter of 500 nm (5.0 wt% dispersed in water with a particle size difference coefficient of about 3%) was purchased from Zhongke Leiming (Beijing, China) Technology Co., LTD. In the beginning, the PS nanospheres dispersed in water/ethanol were slowly driven into the water/air interface through a needle tube and sodium dodecyl sulfate was then added to form a tightly packed monolayer. Then the deionized water was drawn out slowly by a pipette needle until the monolayer was fully contact with the hydrophilic silicon wafer. The successfully assembled PS nanospheres on Si substrate is shown in Figure 1b, and the next step is separated into two different flows: one is for nanopillars and the other is for nanoholes. Firstly, oxygen plasma was used to etch the PS nanospheres for 20 s in order that the size of PS nanospheres met the needs of the experiment for the nanopillars structure, as shown in the Figure 1c. Then, the PS nanospheres can be used as a mask to etch Si materials by the RIE machine with CF_4_ gas with a flow velocity of 65 sccm and a power of 80 W. The nanopillars structures came into being with it, as shown in Figure 1d. Lastly, the oxygen plasma etching technique was used to remove the PS nanospheres on nanopillars (lasting about 2 min). The final nanopillars structures is as shown in Figure 1e. Unlike the 20 s for nanopillars structure, oxygen plasma was used to etch the PS nanospheres for 30 s for the nanoholes structure. Because the PS nanospheres is also etched in the process of Si etching in the Figure 1d. The PS nanospheres should avoid etching too long in the first place for nanopillars in case the PS nanospheres was excessively etched in the end. Then, an Al film with a 50 nm thick formed on the nanoholes structures sample via an evaporation process. This step was using magnetron sputtering with magnetron chamber working below 3 × 10^−6^ Pa, as shown in the Figure 1f. Next, the PS nanospheres soaked in acetone solution that were removed by ultrasound for 3 min. The resulting patterned metal structure can be used as an etching mask for the nanoholes etching, as shown in the Figure 1g. The etching process of Si nanoholes used the same etching parameters as nanopillars by the RIE machine (a CF_4_ gas with a flow velocity of 65 sccm and a power of 80 W). Finally, to remove the Al film, the sample of self-assembled nanoholes was soaked overnight in dilute hydrochloric, as shown in the Figure 1h.

**Figure 2:**
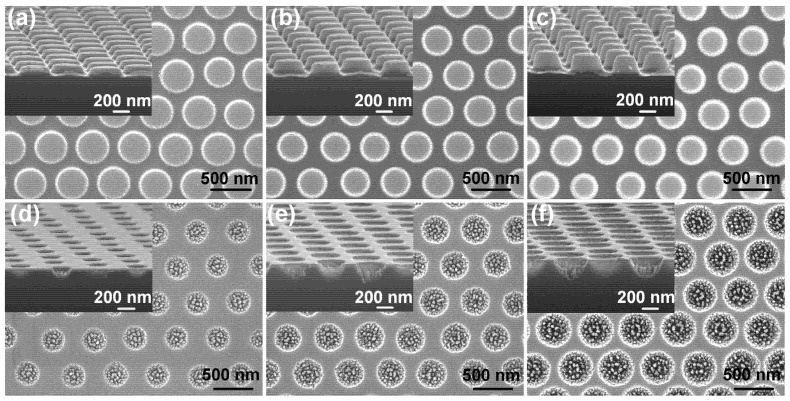
The SEM images for (**a**) top view of nanopillar 1 on Si substrate, (**b**) top view of nanopillar 2 on Si substrate, (**c**) top view of nanopillar 3 on Si substrate, (**d**) top view of nanohole 1 on Si substrate, (**e**) top view of nanohole 2 on Si substrate, (**f**) top view of nanohole 3 on Si substrate (The side view SEM images of each nanostructure as shown in its own inset).

Figure 2a–c show, receptively, the SEM images of three samples of nanopillar 1, nanopillar 2, and nanopillar 3. The side view SEM images of these three samples as shown in its own inset. For comparison, we summarized the size parameters and etching time of nanopillars and nanoholes, as shown in Table 1. The diameter of nanopillars is dependent on the diameter of PS nanospheres, and the height of nanopillars is determined by the etching time of RIE. Because the PS nanospheres were also etched with a slower etching rate when the Si substrate was etching, which leads to the smaller diameter at the top than the bottom of the nanopillars. It is found that the nanopillars are more like truncated cones, as shown in the inset of Figure 2a–c. Similarly, the SEM images of three samples of nanoholes structures as shown in Figure 2d–f, receptively. The side view SEM images of these three samples as shown in its own inset. The Al film was also etched (the etching selectivity ratio of Al to Si is around 1:10) when the Si substrate covered by an Al film is etching. Different from the nanopillars structure, the diameter of nanoholes is increased with the incremental etching time. Therefore, the shape of the nanoholes is a circular funnel, as shown in the inset of Figure 2d–f. One can notice that there are many peaks raised inside of the nanoholes, which was caused by the subtle impurities that failed to clean during the process of the PS nanospheres removal in the experiment. Because the dimension of these peaks is very small compared to wavelength, they can function as an anti-reflection film [24].

### 2.2. Characterizations

The reflectance of silicon nanostructures was measured by ultraviolet-visible Spectrophotometers (UV-VIS, Lambda 1050, PerkinElmer, Waltham, MA, USA) which were equipped with integrating sphere and angle adjusting systems. The surface morphology and cross-sectional images of silicon nanopillars and nanoholes were measured by scanning electron microscope (SEM, JSM-IT500HR, JEOL, Tokyo, Japan). The magnetron sputtering was used to evaporate Al film. The reactive ion etching (RIE) machine was used to etch Si and PS nanospheres.

## 3. Result and Discussion

**Figure 3:**
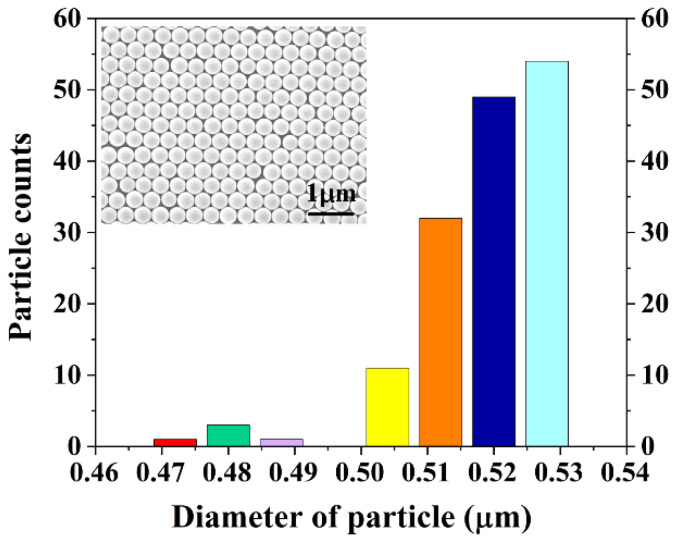
The size distribution of PS nanospheres (The inset is the SEM image of PS nanospheres).

**Figure 4:**
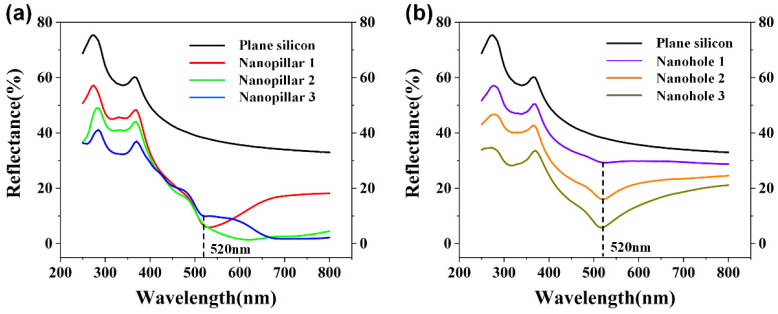
Reflection curves of (**a**) plane silicon and three different sizes for nanopillars samples; (**b**) plane silicon and three different sizes for nanoholes samples.

Figure 3 shows the size distribution and SEM image of PS nanospheres. The period of PS nanospheres is 522 nm, which is the average of distribution data. Figure 4a shows the reflection curves of plane silicon and three different samples of nanopillars in the operation wavelength domain of 250–800 nm. The reflectivity is higher than 30% in short-wavelength region. Moreover, the reflection curves show a sharp drop as the blue-shifts of wavelength. On the other hand, from the nanopillars samples 1 to 3, the rations of nanopillars becoming higher makes the different shapes of nanopillars, which results in the detached reflection curves in the long wavelength region. Figure 4b shows the reflection curves of plane silicon and three different samples of nanoholes in the operation wavelength of our interest. The period of these nanostructures is 522 nm, which is matched with the period of PS nanospheres. It is found that the reflectivity curves of these samples decrease rapidly in the wavelength region of 250 to 520 nm due to the introduction of nanoholes structures on the silicon substrate. At addition, the reflectivity curves of nanoholes structures show the similar trend with the nanopillars structures in the wavelength range from 250 to 520 nm. The reflection dips appear at the wavelength of 520 nm and afterwards the reflectivity increases slowly and levels off at the operation wavelength greater than 520 nm. Furthermore, the reflectivity can further decrease by the optimization of nanostructures parameters. The lowest reflectivity can reach 5.86, 1.42, 1.74, 28.74, 15.99 and 5.83% for nanopillar 1, nanopillar 2, nanopillar 3, nanohole 1, nanohole 2 and nanohole 3, respectively. Due to the same periodic structure, it is also found that reflection dips in the whole nanopillars and nanoholes samples appear at the operation wavelength of 520 nm.

**Figure 5:**
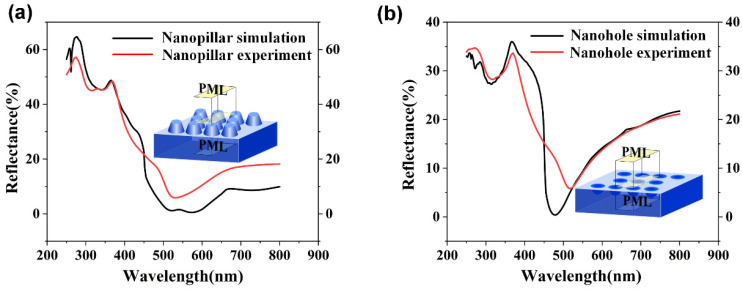
The reflection properties as a function with the operation wavelength in the simulations and experiments for the self-assembled nanopillars structures (**a**), and nanoholes structures (**b**). The inset is the calculation model of nanopillars and nanoholes structures in the 3D FDTD simulation.

**Figure 6:**
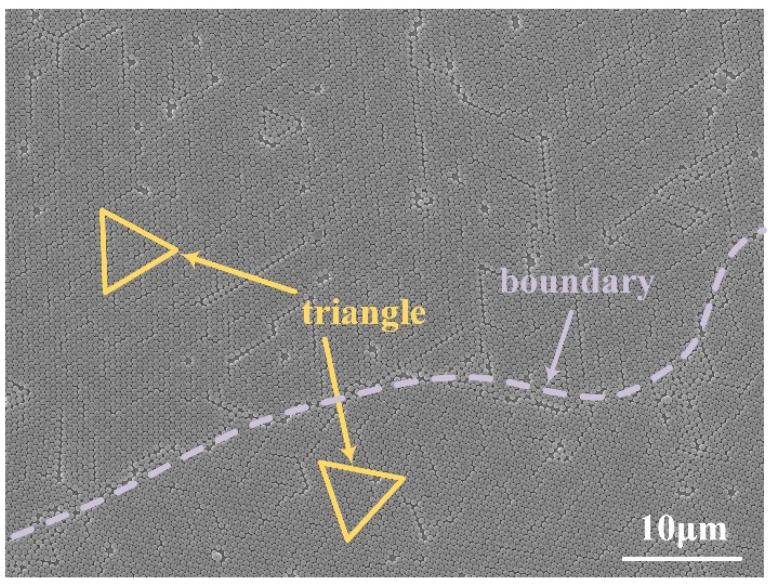
SEM image of the PS nanosphere surface. The red dashed line is the boundary of PS nanospheres region, and the triangle illuminates the arrangement of adjacent nanospheres.

In the following, we numerically investigate and experimentally demonstrate the tunable antireflection properties of self-assembled nanopillars and nanoholes structures. A 3D FDTD method (Lumerical 2020 R2 FDTD solutions software) was used to simulate the unit cell nanostructures that be surrounded by periodic boundary conditions in the x and y directions and the perfectly matched layer absorbing boundary conditions in the z direction. The mesh sizes used for the simulation in x, y and z directions are respectively set to be 8 nm, 8 nm and 5 nm. In the whole simulation, the plane wave source with the operation wavelength of 250–800 nm was used as the experiment source in real environment. The frequency-domain field and power monitor were used to monitor the reflection power of the model. The simulation results are stable as long as the mesh is not larger than 8 nm, 8 nm and 5 nm. The reflection properties of self-assembled nanopillars and nanoholes structures as a function with the operation wavelength in the simulations and experiments, as shown in Figure 5a,b. It can be seen that the simulated data and the experiment data match well in the whole operation wavelength. However, a larger mismatch at the reflectivity dips appears in the simulations and experiments. Because the reflection properties of self-assembled nanopillars and nanoholes structures are very sensitive with the change in nanostructure parameters. On one hand, the fabrication tolerance of nanostructure is inevitable in the experiment, which leads to the larger mismatch at the reflectivity dips in the simulations and experiments. On the other hand, the nanospheres are strictly organized throughout the whole range. On the contrary, the nanospheres had the tendency to form different small high ordered regions in the experiment. The regularities of the nanospheres are different, and the orientation of the triangles formed by adjacent nanospheres are also different for the high ordered region, as shown in the Figure 6. Moreover, the peaks of nanoholes in the experiment also caused less reflection which simulations did not. As this result, the larger mismatch at the reflectivity dips simultaneously occurred in the simulations and experiments.

**Figure 7:**
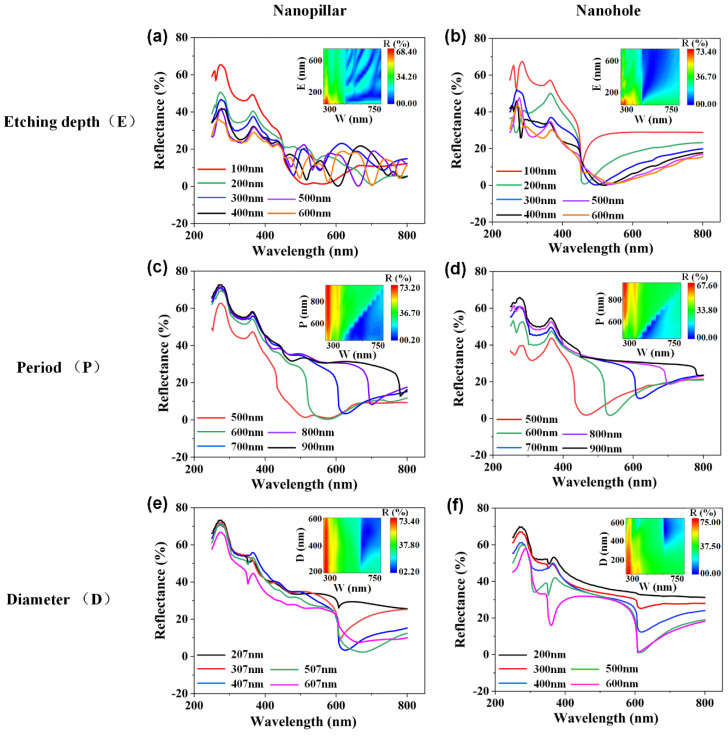
The antireflection properties of the self-assembled nanopillars structures as a function of etching depth (**a**), period (**c**), diameter (**e**). The antireflection properties of the self−assembled nanoholes structures as a function of etching depth (**b**), period (**d**), diameter (**f**). The inset shown the contour plots, which are corresponding to the reflectance curves in the graphs.

In this section, we will focus on discussing the tunable antireflection properties of the self-assembled nanopillars and nanoholes structures with the change in structures parameters using the 3D FDTD method. Figure 7a,b show the reflectance properties of the self-assembled nanopillars and nanoholes structures as a function of the different etching depth (height for nanopillars, depth for nanoholes) of the nanostructure. For the nanopillars structures, it can be clearly seen that the reflectivity decreases with the height of nanopillars in the short-wavelength range. At addition, a sharp dip and oscillation occur at the operation wavelength of 450 nm, which may be caused by the reflected light interference from the Si substrate [21]. For the nanoholes structures, it shows the similar trend of reflectance variation with the nanopillars structures in the short-wavelength range. A sharp dip occurs at the operation wavelength of 450 nm, which is becoming wider with the increased depth the nanoholes structures. Therefore, the reflection of the self-assembled nanopillars and nanoholes structures can reduce with the increase in height and depth. The antireflection properties of the self-assembled nanopillars structure as a function of the nanostructure period, as shown in Figure 7c,d. One can see that the reflectance of the nanopillars and nanoholes structures are increasing with the increase in the nanostructure period in the operation wavelength of 250–800 nm. Due to the diffraction effect, the reflectance dips show a red-shift and gradually increase with the increase in the nanostructure period. As a result, the diffraction intensity of the self-assembled nanostructures is suppressed with the increase in the nanostructure period. Figure 7e,f show the reflectance properties of the self-assembled nanopillars and nanoholes structures with the change in the different diameter. The reflectance is decreasing with the increase in the diameter of nanopillars and nanoholes in the operation wavelength of 250–800 nm. However, the reflectivity increases slowly and levels off at the diameter greater than 700 nm. At addition, it be noticed that the position of reflectance dips depends on the diameter and period of the nanostructures, which can be demonstrated by the grating theory based on the diffraction effect of periodic nanostructures [38,39,40].

In order to understand the operation mechanism of tunable antireflection properties for the self-assembled nanopillars and nanoholes structures with the change in structures parameters. A grating theory can be used to in demonstrations that the position of reflectance dips depends on the nonstructural period. According to the grating theory, the intensity distribution formula of the grating theory can be expressed as follows:

(1) $$I=I_{0}{\left(\frac{\sin\alpha }{\alpha }\right)}^{2}{\left(\frac{\sin N\beta }{\sin\beta }\right)}^{2}$$

where *α* is the radius of the nanostructures and *β* represents the phase-difference between beams of adjacent gratings, which can be expressed by $\alpha =\frac{\pi a\sin\theta }{\lambda }$ and $\beta =\frac{\pi p\sin\theta }{\lambda }$, *I*_0_ = |*E*_0_|^2^ (Intensity of light diffracted by a single slit). *N* is the number of slits. *a* and *p* are the length and period of slits. As shown in Figure 7, the reflection value of lowest points can only be adjusted by the radius and period of the nanostructures, which is corresponding to *a* and *p* in the formulas (1) and (2). Herein, the *I* can reach the maximum value when the period (*p*), the operation wavelength (*λ*), and the diffraction order [*m*, *n*] meet the following formula [41,42]:

(2) $$\left|m\right|,\left|n\right|=\frac{p\sin\theta }{\lambda }<\frac{p}{\lambda };\;\;n,m=0,\pm 1,\pm 2,\cdots$$

where *θ* is diffraction angle. In this work, the period of nanostructures is less than three times of the operation wavelength (*p* < 3 *λ*) in the *x* direction, there are only the reflection orders of 0, ±1 and ±2. However, the third order diffraction should also be considered because there are inconsistent periods in the *x* and *y* directions (*p_y_* = √3 *p_x_*) caused by the 2D hexagonal lattice of nanostructures. Therefore, the diffraction orders of ±1 and ±2 are highly dependent on the operation wavelength, and the *I* shows the negative correlation with the diffraction order. Herein, the reflectance dips will appear when the reflection orders of ±1 occur in the *x* and *y* directions. Moreover, the reflectance dips can only be adjusted by the period of nanostructures, which is consistent with Equation (2). Therefore, the reflectivity dips are caused by the diffraction effect of periodic nanostructures. The tunable anti-reflection property of self-assembled nanopillars and nanoholes structures by the change in structure parameters has a crucial significance for the study of anti-reflection effect and special meaning for deeper understanding about the physical mechanism of nanostructure [43,44]. The introduction of reflectivity dips based on the diffraction effect of periodic nanostructures in this work can be applied to the optical devices with the anti-reflection property. The tunable anti-reflection property by the adjustments of nanostructure parameters is also a meaningful for the anti-reflection of particular wavelength [45].

## 4. Conclusions

In general, the nanopillars and nanoholes structures on silicon substrate successfully obtained using the air–liquid interface self-assembly method based on PS nanospheres, to improve the light absorption of photoelectric devices by the antireflection enhancement. The tunable antireflective properties with the different operation wavelength and nanostructures parameters have been discussed through the FDTD. The operation mechanism of the tunable antireflective property of self-assembled nanopillars and nanoholes structures has been demonstrated by the grating theory based on the diffraction effect of periodic nanostructures. The self-assembled silicon-based nanopillars and nanoholes structures achieved the lowest reflectivity of 1.42% and 5.83% in the operation wavelength of 250-800 nm, which reduced 95.97% and 84.83% respectively compared with the plane silicon. Our study suggests that the self-assembled nanopillars and nanoholes structures are potentially attractive as improving efficiency of photoelectric devices.

## Figures and Tables

**Table 1:** Size parameters and etching time of nanopillars and nanoholes.

Nanostructure-Type	Height (nm)	Period (nm)	Diameter (nm)	Etching Time (s)
Nanopillar 1	107	522	Top:346 Bottom:407	260
Nanopillar 2	152	522	Top:305 Bottom:386	390
Nanopillar 3	207	522	Top:283 Bottom:382	490
Nanohole 1	130	522	276	260
Nanohole 2	202	522	339	390
Nanohole 3	241	522	410	490

## Data Availability

Not applicable.

## References

[1] Dottermusch S., Schmager R., Klampaftis E., Paetel S., Kiowski O., Ding K., Richards B.S., Paetzold U.W. (2019). Micro-cone textures for improved light in-coupling and retroreflection-inspired light trapping at the front surface of solar modules. Prog. Photovolt..

[2] Huang Z., Shi X., Wang G., Leukkunen P., Huttula M., Cao W. (2020). Antireflective design of Si-based photovoltaics via biomimicking structures on black butterfly scales. Sol. Energy.

[3] Liang L., Liu W., Cao Y., Zhu D., Zhang J., Yu Y. (2022). Light trapping PMMA planar ridged waveguide on a laser textured silicon substrate for ultra-low reflectivity. Opt. Laser Technol..

[4] Falcone V., Ballabio A., Barzaghi A., Zucchetti C., Anzi L., Bottegoni F., Frigerio J., Sordan R., Biagioni P., Isella G. (2022). Graphene/Ge microcrystal photodetectors with enhanced infrared responsivity. APL Photonics.

[5] Guan L., Shen G., Liang Y., Tan F., Xu X., Tan X., Li X. (2019). Double-sided pyramid texturing design to reduce the light escape of ultrathin crystalline silicon solar cells. Opt. Laser Technol..

[6] Li H., Hu Y., Wang H., Tao Q., Zhu Y., Yang Y. (2021). Full-Spectrum Absorption Enhancement in a-Si:H Thin-Film Solar Cell with a Composite Light-Trapping Structure. Sol. RRL.

[7] Lu X., Li Y., Lun S., Wang X., Gao J., Wang Y., Zhang Y. (2019). High efficiency light trapping scheme used for ultrathin c-Si solar cells. Sol. Energy Mater. Sol. Cells.

[8] Tang Q., Shen H., Yao H., Gao K., Jiang Y., Li Y., Liu Y., Zhang L., Ni Z., Wei Q. (2019). Superiority of random inverted nanopyramid as efficient light trapping structure in ultrathin flexible c-Si solar cell. Renew. Energy.

[9] Liu X., Li K., Shen J., Gong F. (2021). Hot embossing of moth eye-like nanostructure array on transparent glass with enhanced antireflection for solar cells. Ceram. Int..

[10] Meng L., Shi L., Ge Y., Tang J., Chen Y., Zhong H. (2021). Photon management of combining nanostructural antireflection and perovskite down-shifting composite films for improving the efficiency of silicon solar cells. Sol. Energy Mater. Sol. Cells.

[11] Singh B., Shabat M.M., Schaadt D.M. (2020). Wide angle antireflection in metal nanoparticles embedded in a dielectric matrix for plasmonic solar cells. Prog. Photovolt..

[12] Li W., Li Y.-Q., Shen Y., Zhang Y.-X., Jin T.-Y., Chen J.-D., Zhang X.-H., Tang J.-X. (2019). Releasing the Trapped Light for Efficient Silver Nanowires-Based White Flexible Organic Light-Emitting Diodes. Adv. Opt. Mater..

[13] Lin L., Krahne R., Zaccaria R.P. (2022). Improved Efficiency of Light-Emitting Diodes by Plasmonic Nanopatterning of the Charge-Transfer Layer. Adv. Opt. Mater..

[14] Ye Z.T., Cheng Y.H., Liu K.H., Yang K.S. (2021). Mini-LEDs with Diffuse Reflection Cavity Arrays and Quantum Dot Film for Thin, Large-Area, High-Luminance Flat Light Source. Nanomaterials.

[15] Gu P., Chen J., Yang C., Yan Z., Tang C., Cai P., Gao F., Yan B., Liu Z., Huang Z. (2020). Narrowband Light Reflection Resonances from Waveguide Modes for High-Quality Sensors. Nanomaterials.

[16] He G., Zhang Y.-C., De Tandt C., Stiens J. (2020). A fully electronically tunable millimeter wave lab-in-waveguide nano-fluidic sensor. J. Phys. D Appl. Phys..

[17] Foster J.C., Varlas S., Couturaud B., Coe Z., O'Rei'lly R.K. (2019). Getting into Shape: Reflections on a New Generation of Cylindrical Nanostructures' Self-Assembly Using Polymer Building Blocks. J. Am. Chem. Soc..

[18] Han Z., Jiao Z., Niu S., Ren L. (2019). Ascendant bioinspired antireflective materials: Opportunities and challenges coexist. Prog. Mater. Sci..

[19] Wang W., Qi L. (2019). Light Management with Patterned Micro- and Nanostructure Arrays for Photocatalysis, Photovoltaics, and Optoelectronic and Optical Devices. Adv. Funct. Mater..

[20] Razzaq A., Depauw V., Cho J., Radhakrishnan H.S., Gordon I., Szlufcik J., Abdulraheem Y., Poortmans J. (2020). Periodic inverse nanopyramid gratings for light management in silicon heterojunction devices and comparison with random pyramid texturing. Sol. Energy Mater. Sol. Cells.

[21] Sun T., Tu J., Cao L., Fu T., Li Q., Zhang F., Xiao G., Chen Y., Li H., Liu X. (2020). Sidewall Profile Dependent Nanostructured Ultrathin Solar Cells With Enhanced Light Trapping Capabilities. IEEE Photonics J..

[22] Qiu Y., Wen B., Yang H., Lin Y., Cheng Y., Jin L. (2021). MOFs derived Co@C@MnO nanorods with enhanced interfacial polarization for boosting the electromagnetic wave absorption. J. Colloid Interface Sci..

[23] Kim Y., Gupta P., Kim K. (2020). Controlling the Multiscale Topography of Anodized Aluminum Oxide Nanowire Structures for Surface-Enhanced Raman Scattering and Perfect Absorbers. ACS Appl. Mater. Interfaces.

[24] Sun T., Shui F., Yang X., Zhou Z., Wan R., Liu Y., Qian C., Xu Z., Li H., Guo W. (2022). High Anti-Reflection Large-Scale Cup-Shaped Nano-Pillar Arrays via Thin Film Anodic Aluminum Oxide Replication. Nanomaterials.

[25] Sun T., Shi H., Cao L., Liu Y., Tu J., Lu M., Li H., Zhao W., Li Q., Fu T. (2020). Double grating high efficiency nanostructured silicon-based ultra-thin solar cells. Results Phys..

[26] Shen S., Tang J., Yu J., Zhou L., Zhou Y. (2019). Double-sided and omnidirectional absorption of visible light in tapered dielectric nanostructure coated with non-noble metal. Opt. Express.

[27] Sun T., Liu Y., Tu J., Zhou Z., Cao L., Liu X., Li H., Li Q., Fu T., Zhang F. (2020). Wafer-scale high anti-reflective nano/micro hybrid interface structures via aluminum grain dependent self-organization. Mater. Des..

[28] Li H., Cao L., Fu T., Li Q., Zhang F., Xiao G., Chen Y., Liu X., Zhao W., Yu Z. (2019). Morphology-dependent high antireflective surfaces via anodic aluminum oxide nanostructures. Appl. Surf. Sci..

[29] Kasani S., Curtin K., Wu N. (2019). A review of 2D and 3D plasmonic nanostructure array patterns: Fabrication, light management and sensing applications. Nanophotonics.

[30] Desbiolles B.X.E., Bertsch A., Renaud P. (2019). Ion beam etching redeposition for 3D multimaterial nanostructure manufacturing. Microsyst. Nanoeng..

[31] Zhu T.F., Liang Y., Liu Z.C., Wang Y.F., Shao G.Q., Wen F., Min T., Wang H.X. (2021). Simple way to fabricate orderly arranged nanostructure arrays on diamond utilizing metal dewetting effect. Opt. Express.

[32] Liu T., Tong X., Tian S., Xie Y., Zhu M., Feng B., Pan X., Zheng R., Wu S., Zhao D. (2022). Theoretical modeling of ice lithography on amorphous solid water. Nanoscale.

[33] Jiang Z., Lee B. (2021). Recent advances in small angle x-ray scattering for superlattice study. Appl. Phys. Rev..

[34] Ray D., Wang H.-C., Kim J., Santschi C., Martin O.J.F. (2022). A Low-Temperature Annealing Method for Alloy Nanostructures and Metasurfaces: Unlocking a Novel Degree of Freedom. Adv. Mater..

[35] Liu W., Wang J., Xu X., Zhao C., Xu X., Weiss P.S. (2021). Single-Step Dual-Layer Photolithography for Tunable and Scalable Nanopatterning. Acs Nano.

[36] Xu X., Yang Q., Wattanatorn N., Zhao C., Chiang N., Jonas S.J., Weiss P.S. (2017). Multiple-Patterning Nanosphere Lithography for Fabricating Periodic Three-Dimensional Hierarchical Nanostructures. Acs Nano.

[37] Fang X., Zheng C., Yin Z., Wang Z., Wang J., Liu J., Luo D., Liu Y.J. (2020). Hierarchically Ordered Silicon Metastructures from Improved Self-Assembly-Based Nanosphere Lithography. ACS Appl. Mater. Interfaces.

[38] Naeimi A.A., Darabi E., Mortezapour A., Naeimi G. (2020). Phase-controlled Optical PT symmetry and asymmetric light diffraction in one- and two-dimensional optical lattices. Eur. Phys. J. Plus.

[39] Zhou B., Jia W., Sun P., Wang J., Liu W., Zhou C. (2020). Polarization-independent high diffraction efficiency two-dimensional grating based on cylindrical hole nano arrays. Opt. Express.

[40] Busch K., von Freymann G., Linden S., Mingaleev S.F., Tkeshelashvili L., Wegener M. (2007). Periodic nanostructures for photonics. Phys. Rep..

[41] Gou J., Cansizoglu H., Bartolo-Perez C., Ghandiparsi S., Mayet A.S., Rabiee-Golgir H., Gao Y., Wang J., Yamada T., Devine E.P. (2019). Rigorous coupled-wave analysis of absorption enhancement in vertically illuminated silicon photodiodes with photon-trapping hole arrays. Nanophotonics.

[42] Bonod N., Neauport J. (2016). Diffraction gratings: From principles to applications in high-intensity lasers. Adv. Opt. Photonics.

[43] Agnihotri S.K., Prashant D.V., Samajdar D.P. (2022). A Modified Hexagonal Pyramidal InP nanowire Solar Cell structure for Efficiency Improvement: Geometrical Optimisation and Device Analysis. Sol. Energy.

[44] Baucour A., Kim M., Shin J. (2022). Data-driven concurrent nanostructure optimization based on conditional generative adversarial networks. Nanophotonics.

[45] Jin H., Liu G.L. (2012). Fabrication and optical characterization of light trapping silicon nanopore and nanoscrew devices. Nanotechnology.

